# Training-FreeDetector-to-Promptable-Segmenter Integration for Stacked Cartons: Prompt Accuracy–Efficiency Trade-Offs

**DOI:** 10.3390/s26165229

**Published:** 2026-08-18

**Authors:** Liang Yu, Qi Gao, Shuaiqi Yang, Hao Qiu, Xiaoyan Meng

**Affiliations:** 1 School of Computer and Information Engineering, Xinjiang Agricultural University, Urumqi 830052, China; 320243435@stu.xjau.edu.cn (L.Y.); 320253434@stu.xjau.edu.cn (Q.G.);; 2 Engineering Research Center for Intelligent Agriculture, Ministry of Education, Urumqi 830052, China; 3 Xinjiang Agricultural Informatization Engineering Technology Research Center, Urumqi 830052, China

**Keywords:** instance segmentation, promptable segmentation, detector–segmenter integration, Segment Anything Model, YOLO, stacked cartons, intelligent warehousing

## Abstract

Detector-assisted promptable segmentation can reuse box annotations, but prompt design creates an accuracy–efficiency trade-off in dense scenes. We study a training-free interface in which a detector trained with bounding-box annotations localizes stacked cartons and a frozen segmenter generates masks. Four interfaces are evaluated: Point-Single, Box, ambiguity-aware Point-Max, and Grid-Iter with sparse positive points and one mask-logit feedback pass. On a leakage-audited split, COCO mask AP, fixed-threshold Hungarian-matched metrics, paired bootstrap uncertainty, and synchronized latency were measured. On 773 test images, Box and Grid-Iter achieved mask AP values of 0.8984 and 0.8988. Their paired AP difference was 0.0007 (95% CI, −0.0035 to 0.0047), while matched-mIoU and F1 intervals also included zero. Box required 604.67 ms/image versus 677.33 ms/image for Grid-Iter. A YOLO26s–SAM 3 Box reference achieved a mask AP value of 0.9115 at 320.74 ms/image, whereas mask-supervised YOLOv9c-seg achieved a value of 0.9235 AP on the same cleaned split. The results indicate a metric-dependent Pareto trade-off rather than overall Grid-Iter superiority and position detector-to-segmenter prompting as a reproducible annotation–accuracy option for densely stacked cartons.

## 1. Introduction

Intelligent warehousing has become an important component of smart manufacturing and modern supply-chain management. Reliable perception of packaged goods supports inventory counting, robotic handling, automatic sorting, palletizing, storage-space allocation, and logistics cost estimation. Among commonly handled objects, cartons are particularly important because they appear in a wide range of sizes, textures, colors, and stacking configurations. Accurate carton instance masks can provide not only object counts but also contour information required by downstream tasks such as size estimation, visible-surface measurement, pose analysis, grasp planning, and three-dimensional volume reconstruction. Consequently, robust carton instance segmentation is a fundamental perception problem rather than an isolated image-processing task [1,2,3].

Recent warehouse-oriented vision studies have investigated real-time semantic segmentation of unrecognized objects, stacked-goods positioning in automated storage and retrieval systems, synthetic-data-assisted pallet detection, and deep-learning-based inventory detection for facility-management systems. Together, these studies demonstrate the demand for robust and adaptable visual perception in logistics environments [4,5,6,7].

Stacked-carton scenes are nevertheless difficult for both detection and segmentation algorithms. Adjacent cartons often have similar colors and rectangular appearances, while their boundaries may be partially hidden by mutual occlusion, straps, tape, labels, shadows, or deformation caused by compression. Printed logos and high-contrast textures can attract point-based segmentation models toward local regions instead of the complete carton surface. At the same time, loose detection boxes may contain pixels from neighboring cartons, and a single carton may be split into several visible fragments. These factors make it insufficient to evaluate a method only by object detection accuracy. For warehouse measurement applications, the predicted masks must also maintain instance separation, contour adherence, and visible-region completeness under dense stacking.

Traditional carton-perception pipelines frequently rely on thresholding, edge extraction, handcrafted geometric features, and morphological processing. Such methods can work in controlled scenes but are sensitive to illumination variation, cluttered backgrounds, deformation, and changes in carton appearance. Fully supervised instance-segmentation models provide a more robust alternative and have been applied to unknown stacked objects, carton datasets, and synthetic-to-real warehouse perception [3,8,9]. However, these models generally require pixel-level polygon masks for training. Compared with bounding boxes, instance masks are considerably more laborious to annotate, especially in scenes containing many mutually occluded cartons. The annotation burden also makes frequent model updates difficult when a warehouse introduces new packaging types, camera viewpoints, or lighting conditions.

The Segment Anything Model (SAM) offers a different route through prompt-driven segmentation and strong cross-domain generalization [10]. Instead of training a domain-specific mask predictor from scratch, SAM can generate masks from points, boxes, or coarse mask prompts. This property has motivated a growing number of detector-assisted SAM frameworks in medical imaging, agriculture, road analysis, industrial inspection, and robotic perception [11,12,13,14,15,16,17]. In these systems, an object detector first localizes the target and then supplies a spatial prompt to SAM. Although this detector–segmenter cascade reduces the need for task-specific mask fine-tuning, its final performance remains strongly dependent on how the detector output is converted into a prompt. Automatic prompt-generation studies have also shown that bounding boxes derived from weak localization cues can guide SAM when dense mask annotations are scarce, while box–mask consistency can be used to verify candidate objects and annotations [18,19].

More specifically, YOLO–SAM combinations have been explored for crop-disease analysis and crop detection, anomalous traffic-sign segmentation, aerial power-line inspection, remote-sensing parcel extraction, and aquatic-animal monitoring [20,21,22,23,24,25]. Related work has also extended detector-guided SAM to vision-based robotic disassembly [26], while recent studies have investigated explicit alignment between YOLO detections and SAM masks [27]. These studies demonstrate that detectors and promptable segmenters can be connected in many domains. Accordingly, the novelty claimed here is not the juxtaposition of YOLOv9 and SAM, but the controlled study of the information, failure modes, accuracy, and cost of their prompt interfaces for densely stacked homogeneous objects.

Prompt design is especially important for stacked-carton instance segmentation. A center-point prompt is computationally simple, but it provides no explicit information about target extent and may activate a local texture, tape region, or only one visible fragment. Direct box prompting supplies stronger spatial coverage, yet pixels belonging to neighboring cartons may be included when boxes are loose or overlap. Dense point prompts potentially distribute positive evidence across the visible carton region, but excessively dense sampling increases the chance of placing prompts on occlusion boundaries or adjacent instances. Moreover, SAM’s ambiguity-aware multi-mask output and iterative mask-input mechanism create additional design choices: candidate masks can be geometrically screened, and previous logits can be fed back to refine boundaries. Existing detector–SAM studies often select one prompt type for a particular application, whereas systematic evidence regarding the relative behavior, parameter sensitivity, computational cost, and actual contribution of these prompt mechanisms remains limited for densely stacked cartons [28,29,30].

To address this problem, this study formulates stacked-carton segmentation as a training-free detector-to-promptable-segmenter integration problem. “Training-free” refers specifically to the interface and frozen segmenter: the detector is still fine-tuned from carton boxes, and ground-truth masks are retained for validation and evaluation. YOLOv9-C first supplies category-specific instance locations; SAM ViT-H then produces contours from four interfaces. Point-Single tests the minimum-information center point. Box passes the detected extent directly and is treated as the primary deployment baseline. Point-Max tests whether ambiguity-aware candidate selection can rescue an under-specified point. Grid-Iter tests whether distributed positive evidence and one mask-logit feedback decode improve visible-region completeness enough to justify their additional cost. The validation-selected Grid-Iter uses a 2×2 inset grid in the final test.

Because YOLOv9 and the first-generation SAM are no longer current [31], we retain them only to reproduce and analyze the submitted controlled experiment. We additionally evaluate the portable Box interface with YOLO26s and SAM 3 [32,33]. This modern reference is reported with its model-native preprocessing and precision and is not treated as a capacity-matched single-variable ablation.

The main contributions of this work are summarized as follows:

- We precisely formulate four detector-to-segmenter interfaces and connect Point-Max and Grid-Iter to testable hypotheses about point ambiguity, spatial extent, positive-prompt contamination, and iterative mask feedback.
- We introduce a common evaluation protocol with cross-split duplicate auditing, low-score COCO mask AP, Hungarian one-to-one matching, fixed-threshold instance metrics, paired image-level bootstrap intervals, and synchronized component timing.
- Validation-only selector, grid-density, iteration, buffer, and morphology studies separate the contributions of the proposed heuristics. Test-set results are interpreted by an accuracy–latency Pareto rule, with Box preferred when accuracy differences are practically and statistically indistinguishable.
- A YOLO26s–SAM 3 Box reference evaluates whether the main interface conclusion transfers to newer detector and promptable-segmenter generations while explicitly disclosing model-native differences.
- Common-split Mask R-CNN, YOLOv8n-seg, and YOLOv9c-seg references place the annotation-efficient detector–promptable-segmenter interface in context without concealing the supervised models’ additional polygon-mask training requirement.

The remainder of this paper is organized as follows. Section 2 introduces the YOLOv9–SAM framework, the Point-Max and Grid-Iter strategies, the dataset, and the evaluation metrics. Section 3 presents the quantitative comparison, runtime analysis, parameter sensitivity, qualitative results, and ablation study. Section 4 discusses the behavior of different prompts, the accuracy–efficiency trade-off, practical limitations, and future extensions. Finally, Section 5 concludes the study.

## 2. Materials and Methods

### 2.1. System Scope and Training-Free Integration

The controlled system contains a box detector and a frozen promptable segmenter. YOLOv9-C is the official configuration defined by models/detect/yolov9-c.yaml in the official YOLOv9 repository (github.com/WongKinYiu/yolov9) [34]; the suffix “C” is a configuration name and is not expanded as “compact.” The detector is initialized from the official COCO checkpoint and fine-tuned using carton bounding boxes. SAM ViT-H [10] is loaded from its official checkpoint and remains frozen throughout every carton experiment. Neither predicted nor ground-truth carton masks update SAM’s parameters.

At inference, the detector produces $B=\{\left(x_{1},y_{1},x_{2},y_{2},c,s\right)\}$, where $\left(x_{1},y_{1},x_{2},y_{2}\right)$ is a clipped box in image coordinates, *c* is the carton class, and *s* is its confidence. A deterministic interface converts each box into a point, box, point-candidate set, or grid-plus-mask prompt. Detector and segmenter are integrated because their capabilities are complementary: inexpensive box supervision supplies category-specific instance localization, whereas the frozen promptable segmenter supplies class-agnostic contours. “Training-free” therefore describes the interface and segmenter adaptation, not detector training or the evaluation process. The cascade cannot recover detector misses, and loose boxes can propagate neighboring pixels into the masks.

### 2.2. Prompt Interfaces and Their Rationale

**Figure 1:**
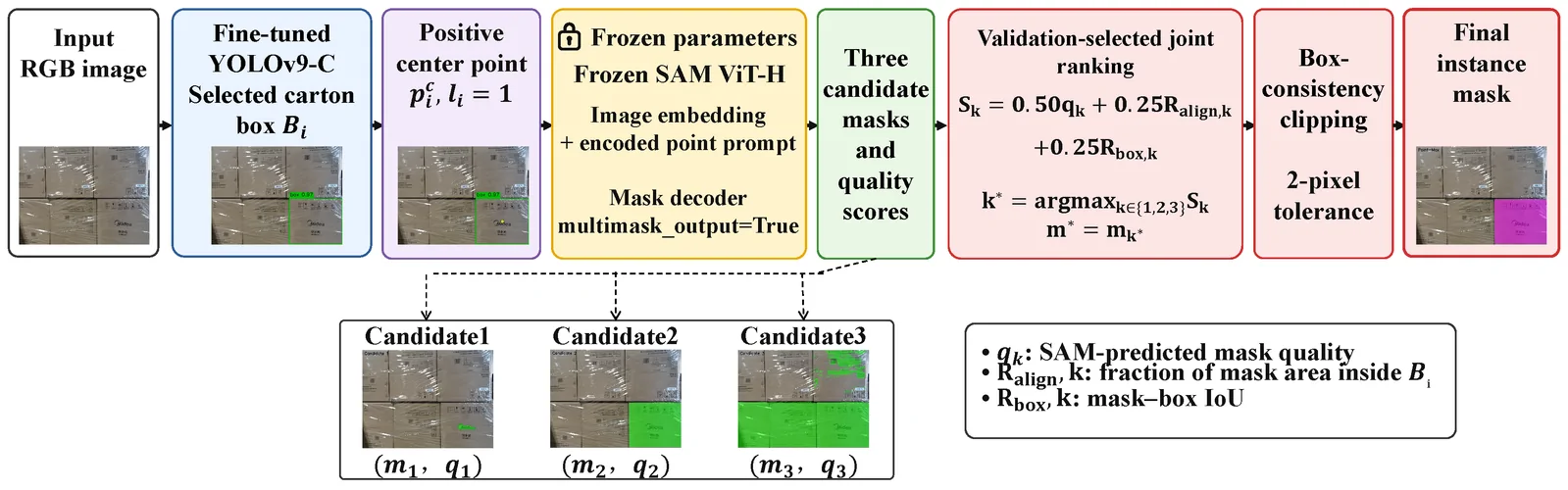
Executed Point-Max interface. Fine-tuned YOLOv9-C supplies the selected carton box $B_{i}$, whose center is encoded as one positive point. Frozen SAM ViT-H returns three ambiguity-aware masks and their predicted quality scores. The candidates are ranked by the validation-selected joint score in Equation (2); the highest-ranked mask is then clipped for box consistency using a two-pixel tolerance. Colors are used only to distinguish processing stages and do not encode quantitative values.

**Figure 2:**
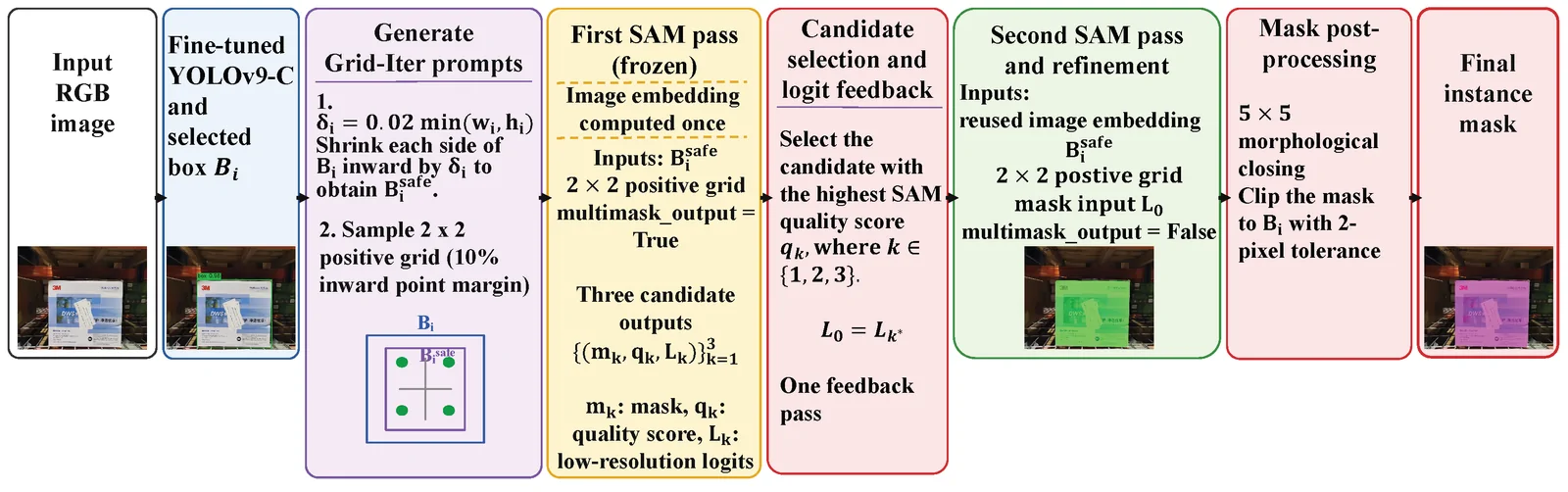
Executed Grid-Iter interface. Each side of the selected detector box $B_{i}$ is moved inward by $0.02\min(w_{i},h_{i})$ to form $B_{i}^{\mathrm{safe}}$, and a 2×2 positive grid is sampled with a 10% inward point margin. In the first frozen SAM pass, the safe box and grid produce three candidate masks, quality scores, and low-resolution logits. The logits of the highest-quality candidate are fed back once for a second decode. The resulting mask undergoes 5×5 morphological closing and is clipped to $B_{i}$ using a two-pixel tolerance. Colors are used only to distinguish processing stages and do not encode quantitative values.

The same detections and image embeddings are reused across the four interfaces. Point-Single places one positive point at the box center and is the minimum-information baseline. Box passes the clipped detector box directly to SAM and is the primary deployment baseline. Point-Max tests whether ambiguity-aware candidate selection compensates for a point prompt that does not encode extent. Grid-Iter tests whether distributed foreground evidence and one mask-feedback decode improve visible-region coverage enough to justify their extra cost. Figure 1 and Figure 2 show the two multi-stage interfaces.

For Point-Single and Box, multimask_output=False; SAM returns one mask and no external two-pixel clipping, morphology, or SAM-score threshold is applied. Point-Single supplies only the positive center point, whereas Box supplies only the detector box. Point-Max supplies only the center point with multimask_output=True, ranks all three candidates by the joint score below, and clips the chosen mask to the detector box with a two-pixel tolerance. Grid-Iter supplies both the 2%-shrunk box and positive grid points. Its first decode enables multimask output and selects the candidate with the highest SAM quality score; the selected low-resolution logits are supplied to one second decode with multimask output disabled. Only Grid-Iter applies 5-pixel morphological closing followed by two-pixel-tolerance box clipping. SAM quality is multiplied by detector confidence for COCO ranking, but no separate SAM-quality cutoff is used.

**Figure 3:**
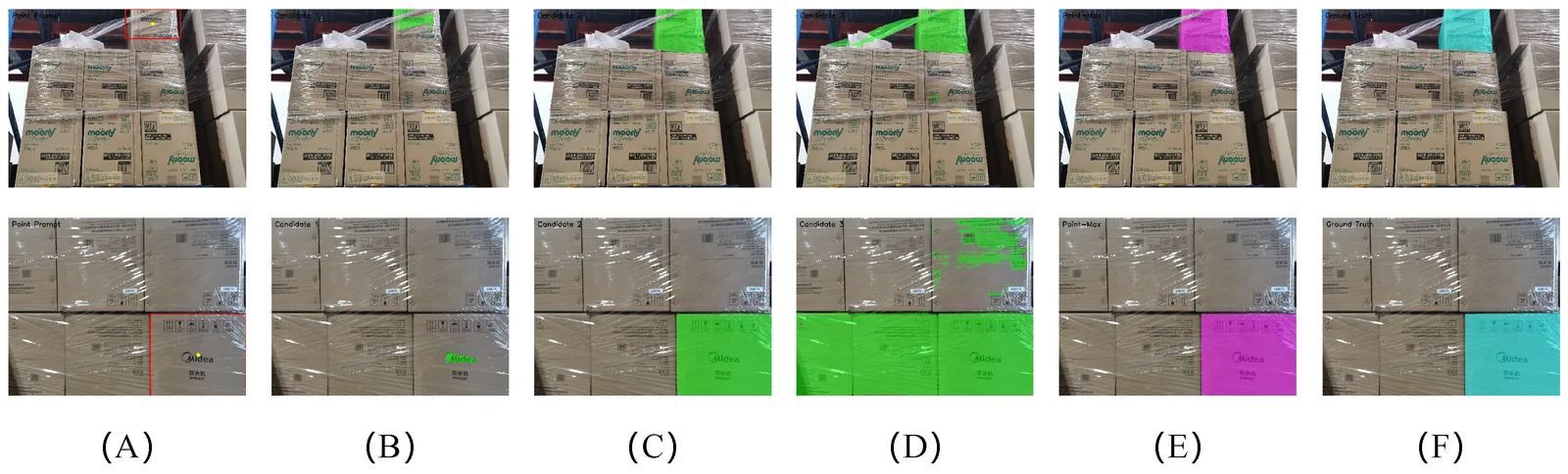
Point-Max, with all panels identified: (**A**) detector box and center point; (**B**) the first candidate mask; (**C**) the second candidate mask; (**D**) the third candidate mask; (**E**) the selected Point-Max prediction; and (**F**) the ground-truth annotation.

**Figure 4:**
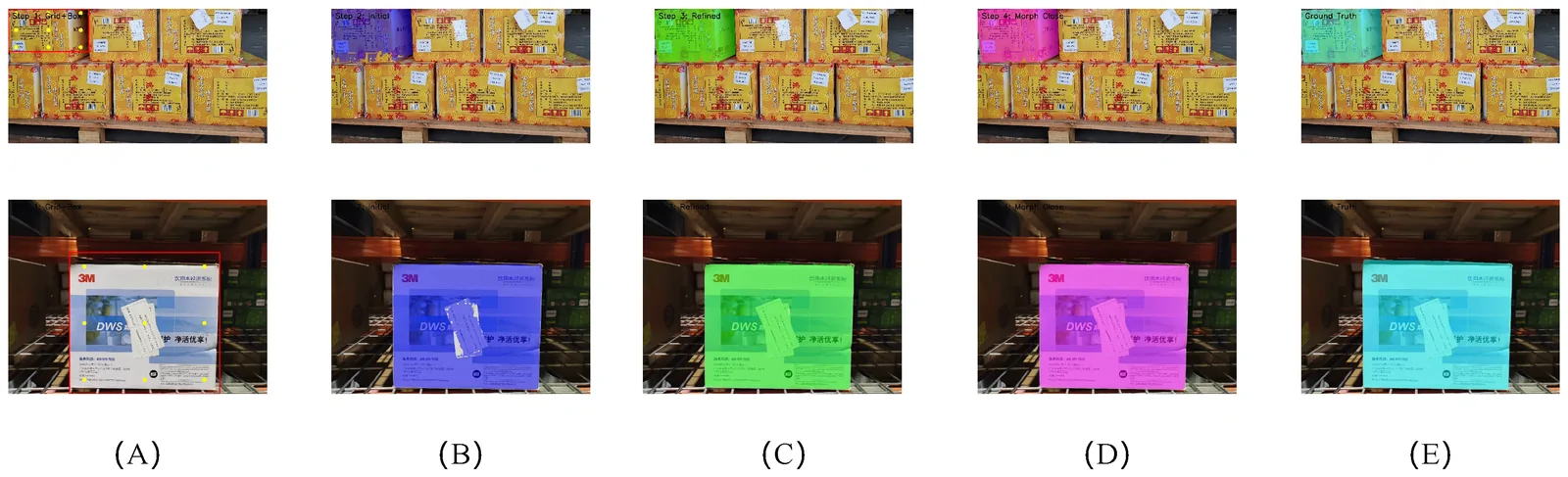
Grid-Iter, with all panels identified: (**A**) detector box and inset positive grid; (**B**) first-pass mask; (**C**) refined prediction after one mask-logit feedback pass; (**D**) final prediction after morphological closing and box-consistency clipping; and (**E**) the ground-truth annotation.

The two proposed multi-stage prompting interfaces are specified as follows:

- Point-Max Strategy (Figure 3): The box center activates SAM’s three-mask ambiguity output. Five selector families were compared on validation data: seeded random, SAM score, box intersection, box coverage, and a joint quality–geometry score. The final Point-Max test ranks all candidates by the joint score. Alignment, area, and centroid validity constraints belong only to the separately evaluated geometric-maximum comparator. This design tests a selector hypothesis; it does not assume that the largest candidate is intrinsically correct.
- Grid-Iter Strategy (Figure 4): All grid points are labeled foreground, so denser sampling can place invalid positive prompts on an occluder, gap, or neighboring carton. Grid sizes 2, 3, 5, and 7 were therefore compared on validation data. The selected 2×2 grid and a 2%-shrunk box are supplied jointly to SAM; the points use a 10% inward margin. One logit-feedback decode is followed by 5-pixel closing and clipping to the original detector box with a 2-pixel tolerance. Grid-No-Iter and Grid-Iter-No-Morph isolate the extra operations.

All selector and grid configurations were frozen from validation data before the final test. Mask AP was the primary validation criterion; when differences were small, latency and interface complexity were used as secondary decision criteria. At the final comparison stage, paired uncertainty and latency are reported together rather than using a fourth-decimal metric difference to declare an overall winner. Accordingly, Grid-Iter is a conditional accuracy-oriented alternative rather than a presumed overall winner.

Ultimately, the pipeline outputs pixel-wise binary masks aligned with the original image resolution, which can be directly utilized for quantitative evaluation and downstream warehouse perception tasks. The detailed procedures of the Point-Max and Grid-Iter interfaces are summarized in Algorithms 1 and 2, respectively.
**Algorithm 1** Point-Max with Validation-Fixed Joint Candidate Selection**Input**: YOLOv9 detected bounding box $B_{i}$, SAM pre-trained model**Output**: Final optimal carton instance mask $m^{*}$  1:  Calculate geometric center point $p_{c}$ of bounding box $B_{i}$  2:  Feed $p_{c}$ into SAM and generate candidate mask set $M=\{m_{1},m_{2},m_{3}\}$  3:  **for all** $m_{k}\in M$ **do**  4:      Compute spatial alignment ratio $R_{align}=\mathrm{Area}\left(m_{k}\cap B_{i}\right)/\mathrm{Area}\left(m_{k}\right)$  5:      Compute box IoU $R_{box}=\mathrm{Area}\left(m_{k}\cap B_{i}\right)/\mathrm{Area}\left(m_{k}\cup B_{i}\right)$  6:      Compute joint score $S_{k}=0.50q_{k}+0.25R_{align}+0.25R_{box}$  7:  **end for**  8:  Select the candidate with maximum joint score as $m^{*}$  9:  Clip the selected mask using the two-pixel-tolerance box: $M_{PM}=m^{*}\cap B_{buffer}$10:  **return** $M_{PM}$

**Algorithm 2** Grid-Iter Iterative Refinement Prompt Strategy
**Input**: YOLOv9 detected bounding box $B_{i}$, SAM model, structuring element *K***Output**: Complete refined carton mask $M_{final}$  1:  Shrink $B_{i}$ by 2% of its shorter side to obtain $B_{i}^{safe}$  2:  Sample the validation-selected 2×2 positive grid $P_{grid}$ inside $B_{i}^{safe}$ with 10% inward margin  3:  Initial multimask inference: $\left\{M_{k}^{\left(0\right)},L_{k}^{\left(0\right)},q_{k}\right\}=F_{SAM}\left(B_{i}^{safe},P_{grid},Ø\right)$  4:  Select $k^{*}=\arg\max_{k}q_{k}$  5:  Logit feedback: $M^{\left(1\right)},L^{\left(1\right)}=F_{SAM}\left(B_{i}^{safe},P_{grid},L_{k^{*}}^{\left(0\right)}\right)$  6:  Execute morphological closing operation: $M_{close}=\left(M^{\left(1\right)}⊕K\right)⊖K$  7:  Truncate mask within buffered bounding box $B_{buffer}$  8:  Obtain final segmentation mask $M_{final}=M_{close}\cap B_{buffer}$  9:  **return** $M_{final}$


### 2.3. Proposed Prompt Engineering Strategies

To systematically address the challenges of severe occlusion and boundary overlap inherent in carton stacking scenarios, we mathematically formalize our two optimized prompting mechanisms.

#### 2.3.1. Point-Max Strategy

For a given bounding box $B_{i}=\left(x_{min},y_{min},x_{max},y_{max}\right)$ predicted by YOLOv9, the geometric center prompt $p_{c}$ is derived as:

(1) $$p_{c}=\left(\frac{x_{min}+x_{max}}{2},\frac{y_{min}+y_{max}}{2}\right)$$

By feeding $p_{c}$ into SAM with multimask output enabled, a set $M={\left\{\left(m_{k},q_{k}\right)\right\}}_{k=1}^{3}$ is generated, where $q_{k}$ is SAM’s predicted mask quality. For each candidate, alignment $R_{align}$, mask-to-box area ratio $R_{area}$, and box IoU $R_{box}$ are computed. The validation-selected joint selector is

(2) $$m^{*}=\underset{m_{k}\in M}{\arg\max}\left(0.50q_{k}+0.25R_{align,k}+0.25R_{box,k}\right),$$

(3) $$R_{align,k}=\frac{|m_{k}\cap B_{i}|}{|m_{k}|},\;R_{area,k}=\frac{|m_{k}|}{|B_{i}|},\;R_{box,k}=\frac{|m_{k}\cap B_{i}|}{|m_{k}\cup B_{i}|}.$$

For the geometric-max comparator, a candidate is valid when $R_{align}\geq 0.6$, $0.05\leq R_{area}\leq 1.5$, and its centroid lies inside a box shrunk by 2% of its shorter side. The joint selector ranks all candidates by the displayed score; the validity rules are retained as a separately reported selector rather than silently conflated with the joint score. A 2-pixel tolerance box is used for final clipping:

(4) $$M_{PM}=m^{*}\cap B_{buffer}$$

#### 2.3.2. Grid-Iter Strategy

For heavily occluded instances, a single point may provide insufficient spatial guidance. Grid-Iter first shrinks the detector box by 2% of its shorter side to obtain $B_{i}^{safe}$ and then samples an n×n positive grid $P_{grid}$ using a 10% inward point margin. The safe box and positive points are supplied jointly to SAM. Validation experiments considered n∈{2,3,5,7} and selected n=2 before the test set was opened. Let $F_{SAM}$ denote the SAM mask decoder, which takes the safe box, prompt points, and an optional prior mask-logit tensor as inputs. The first decode returns three masks and their quality scores; the highest-scoring candidate $k^{*}$ supplies one feedback decode:

(5) $$\begin{array}{cc} \left\{M_{k}^{\left(0\right)},L_{k}^{\left(0\right)},q_{k}\right\} & =F_{SAM}\left(B_{i}^{safe},P_{grid},Ø\right),\;k^{*}=\arg\max_{k}q_{k} \end{array}$$

(6) $$\begin{array}{cc} M^{\left(1\right)},L^{\left(1\right)} & =F_{SAM}\left(B_{i}^{safe},P_{grid},L_{k^{*}}^{\left(0\right)}\right) \end{array}$$

where *M* is the thresholded mask and *L* is SAM’s low-resolution mask-logit output. This operation supplies a second decoder input but does not by itself prove boundary recovery or causal attention changes; its aggregate contribution is measured by the no-iteration ablation. We therefore treat logits only as an algorithmic input and do not interpret their visualization as evidence about SAM’s internal attention or a causal mechanism.

To test whether a simple local post-processing step changes aggregate masks, a morphological closing operation (dilation ⊕ followed by erosion ⊖) is applied using a 5×5 structuring element *K*:

(7) $$M_{close}=M^{\left(1\right)}•K=\left(M^{\left(1\right)}⊕K\right)⊖K$$

Ultimately, to strictly confine the segmentation within the instance boundary, a spatial truncation is performed using an expanded bounding box $B_{buffer}$ (with a 2-pixel tolerance margin), yielding the final instance mask:

(8) $$M_{final}=M_{close}\cap B_{buffer}$$

**Figure 5:**
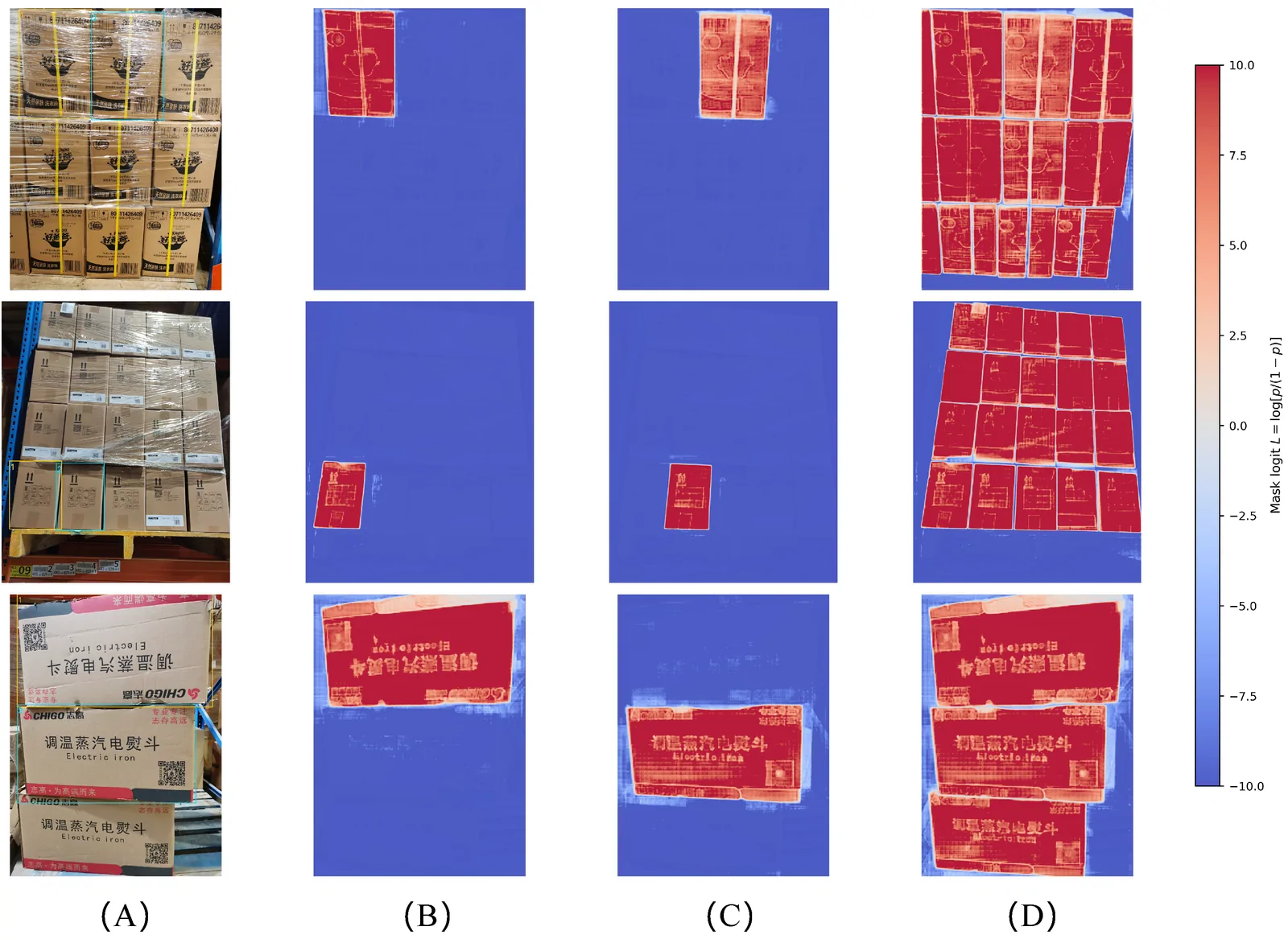
Initial SAM mask-logit visualization for three representative scenes. (**A**) RGB images with two numbered target boxes; (**B**,**C**) instance-specific initial logits for targets 1 and 2; and (**D**) the pixelwise maximum over detected-instance logit maps. All heatmaps share the coolwarm colormap and display interval [−10,10]; values outside the interval are clipped only for display. The color bar reports raw mask logit L=log[p/(1−p)], where p=sigmoid(L); the values are not interpreted as calibrated probabilities.

Figure 5 visualizes the raw initial mask logits used by the feedback interface. If *L* denotes a mask logit, its corresponding sigmoid output is p=sigmoid(L), equivalently L=log[p/(1−p)]. The displayed values are therefore not treated as calibrated probabilities. A single perceptually uniform diverging colormap and a shared [−10,10] interval are used for all panels; values outside that interval are clipped only for visualization. The zero level is the decoder’s binary decision boundary. The figure is descriptive evidence about the signal supplied to the second decode, not proof of an internal attention mechanism or a causal explanation for the final mask.

### 2.4. Dataset and Data Split

All experiments use the live-captured portion of the public Stacked Carton Dataset (SCD) [1]. We use a fixed-seed image-level 8:1:1 allocation because approximately 80% of the data are retained for detector fitting while equally sized validation and test subsets support parameter selection and a held-out final assessment. This ratio is an experimental design choice: changing the training, validation, and test proportions can change both model fitting and uncertainty. Two recent fire-door text-classification studies, for example, used a 60:20:20 design [35,36]; they demonstrate that alternative allocations are reasonable but do not establish a universal optimum for image segmentation.

We audited the original allocation before retraining. SHA-256 identified exact matches, and candidate near duplicates were generated by 64-bit difference hashing (Hamming distance ≤5) and retained only when normalized resized-pixel mean absolute difference was ≤0.08. All 15 initially flagged cross-split pairs were manually confirmed as leakage; redundant members and their paired annotations were removed, followed by a second audit and manual check. The final detector split contains 6171 training, 773 validation, and 773 test images. Re-audit found zero exact and zero perceptual near-duplicate cross-split pairs. A filename-only trailing-frame heuristic still produced two shared group identifiers; because the public files do not provide an authoritative acquisition-scene map, these identifiers are reported as a residual limitation rather than claimed as confirmed scene leakage. Future releases should include source-scene metadata and allocate complete sequences as groups.

Bounding boxes are used to train the detector. Polygon masks are used for validation-only prompt selection, final evaluation, paired statistics, and the post hoc grid-purity analysis; they are not supplied to SAM during inference and never update SAM’s weights.

### 2.5. Model Training and Reproducibility

YOLOv9-C was initialized from yolov9-c.pt and trained for 100 epochs at 640×640 resolution with batch size 16, rectangular batches, eight data-loading workers, full-RAM caching, seed 42, and early-stopping patience 100. The optimizer was SGD with initial learning rate 0.01, final factor 0.01, a linear rather than cosine schedule, momentum 0.937, weight decay $5\times 10^{-4}$, and three warm-up epochs. Augmentation settings were HSV gains (0.015,0.7,0.4), translation 0.1, scaling 0.9, horizontal flip 0.5, mosaic 1.0, mixup 0.15, and copy–paste 0.3; rotation, shear, perspective, and vertical flip were zero. The checkpoint with the best repository-defined validation fitness (best.pt) was used. Training on one NVIDIA A10G 24 GB GPU (NVIDIA Corporation, Santa Clara, CA, USA) hosted on the Zhisuanyunfei cloud platform in China required 5.338 h. The software environment was Python 3.10.14, PyTorch 2.1.2+cu121, CUDA 12.1, and cuDNN 8.9.2. The supplied source copy lacked .git metadata; therefore, we do not report an unverifiable commit identifier.

The modern reference uses YOLO26s initialized from its official checkpoint and trained for 100 epochs at 640×640, batch size 16, seed 42, and eight workers. Ultralytics 8.4.112 resolved optimizer=auto to AdamW (initial learning rate 0.002, momentum 0.9, weight decay $5\times 10^{-4}$). Training used Python 3.12.13, PyTorch 2.10.0+cu128, and the same A10G and required 1.932 h. SAM 3 was frozen.

The supervised references use the same cleaned image split and polygon labels. YOLOv8n-seg and YOLOv9c-seg were initialized from their official pretrained checkpoints and trained for 100 epochs at 640×640 using deterministic seed 42, SGD, initial learning rate 0.01, final factor 0.01, momentum 0.937, weight decay $5\times 10^{-4}$, three warm-up epochs, AMP, and mosaic disabled during the final 10 epochs. Their model-appropriate resource settings were batch size 8 with eight workers and batch size 24 with six workers, respectively; training required 2.601 and 4.320 h. Mask R-CNN ResNet-50-FPN-v2 was initialized from the Torchvision COCO checkpoint and trained for 24 epochs with batch size 2, six workers, SGD (learning rate 0.005, momentum 0.9, weight decay $5\times 10^{-4}$), milestones at epochs 16 and 22 with factor 0.1, AMP, and horizontal flipping with probability 0.5. Validation COCO AP was checked every four epochs; epoch 24 was selected and total training time was 11.951 h. The supervised runs used Python 3.12.13, PyTorch 2.10.0+cu128, Torchvision 0.25.0+cu128 where applicable, and the same A10G. These are common-data and common-evaluation references, not identical-optimizer ablations; model-specific batch sizes and schedules are disclosed rather than artificially forced to match.

### 2.6. Archived Release and Code Traceability

The exact revision artifact is archived as version 1.0.0 on Zenodo at https://doi.org/10.5281/zenodo.21860345 [37]. The archive contains the frozen source code, launch scripts, configurations, cleaned split manifests, derived COCO annotations, author-trained checkpoints, prediction files, per-image timing records, and statistical outputs used in this manuscript. Its ZIP SHA-256 digest is 5129d87caab462c14b63116d0a4c88468fe3be15ef56c31af7ad1ed5faad42fd; an internal SHA256SUMS.txt records file-level digests. This versioned DOI and checksum identify the executed code release despite the unavailable historical Git metadata. Dataset images and third-party foundation-model checkpoints are not redistributed; their official sources, versions, and available integrity identifiers are documented in the archive.

### 2.7. Evaluation Metrics

Detector inputs use letterbox resizing to 640×640. Predicted boxes are inverse-transformed to image coordinates before prompting. Images whose long side exceeds 1024 pixels are uniformly reduced for SAM ViT-H; SAM applies its own resize transform, and every returned mask is restored to the annotation resolution before encoding and evaluation. The modern SAM 3 reference uses its model-native 1008-pixel input. These model-native sizes are disclosed because the cross-generation experiment is not a single-variable ablation.

To avoid conflating contour quality with instance coverage, the experiments report two complementary groups of metrics. First, COCO-style mask AP is computed over IoU thresholds from 0.50 to 0.95 in steps of 0.05. YOLO non-maximum suppression uses IoU 0.5. Predictions are retained from detector confidence 0.001, with at most 300 detections per image; pre-filtering at 0.5 is avoided because it truncates the precision–recall curve. We report mask AP, AP_50_, AP_75_, and AR_100_. Mask confidence is the detector confidence multiplied by SAM’s predicted mask-quality score.

Second, a fixed-threshold one-to-one instance matching evaluation is performed at mask IoU ≥0.5. A matched prediction is counted as a true positive (TP); unmatched predictions and ground-truth instances are counted as false positives (FP) and false negatives (FN), respectively. The instance-level mean IoU is computed over matched mask pairs:

(9) $$\mathrm{Instance}\text{-}\mathrm{level}\;\mathrm{mIoU}=\frac{1}{N_{TP}}\sum_{i=1}^{N_{TP}}\frac{|M_{\mathrm{pred}}^{\left(i\right)}\cap M_{\mathrm{gt}}^{\left(i\right)}|}{|M_{\mathrm{pred}}^{\left(i\right)}\cup M_{\mathrm{gt}}^{\left(i\right)}|}.$$

Precision, Recall, and F1-score are defined as

(10) $$\mathrm{Precision}=\frac{TP}{TP+FP}$$

(11) $$\mathrm{Recall}=\frac{TP}{TP+FN}$$

(12) $$F1=\frac{2\cdot \mathrm{Precision}\cdot \mathrm{Recall}}{\mathrm{Precision}+\mathrm{Recall}}.$$

Predictions and annotations at the fixed detector-score operating point of 0.5 are paired within each image by Hungarian maximum-IoU assignment. Assigned pairs below mask IoU 0.5 are rejected; unmatched predictions and annotations become false positives and false negatives. The mIoU therefore measures boundary quality only among accepted one-to-one matches, whereas AP and F1 additionally penalize missed and duplicated instances.

Paired uncertainty uses the image as the bootstrap unit: 10,000 replicates for fixed-threshold metrics and 1000 replicates for COCO AP. We report the Grid-Iter-minus-Box difference, percentile 95% confidence interval, and bootstrap probability that Grid-Iter exceeds Box. Cohen’s paired $d_{z}$ is reported for decomposable matched metrics and latency, but not for nonlinear dataset-level COCO AP.

Latency was measured on the A10G after 50 untimed warm-up images and three complete sequential trials for each YOLOv9-C/SAM ViT-H strategy. CUDA synchronization bracketed detector inference, SAM encoding, mask decoding, coordinate post-processing, and strategy logic. CPU–GPU transfers and model-internal tensor preprocessing occurring inside those timed calls are included; external image-file loading, COCO evaluation, result serialization, and visualization are excluded. Mean, sample standard deviation, median, P95, component means, and peak allocated CUDA memory are reported. The modern reference used the same test images and five trials, but FP16, a 1008-pixel SAM 3 input, and batched box decoding; its speed is therefore a system reference, not an architecture-only comparison.

## 3. Results

### 3.1. YOLOv9 Object Detection Performance

The cleaned-split detector completed all 100 epochs in 5.338 h. At the final training validation pass it obtained precision 0.970, recall 0.964, mAP_50_ 0.989, and mAP_50:95_ 0.940. Table 1 reports the held-out test detections using COCO evaluation with confidence 0.001. Detection and mask results are separated because detector recall bounds the downstream cascade.

### 3.2. Segmentation Performance of Different Prompting Strategies

All YOLOv9-C/SAM ViT-H strategies were rerun on the same NVIDIA A10G with the same 773 test images and detector outputs. Validation-only sweeps selected the Point-Max joint selector and the 2×2 Grid-Iter configuration before test evaluation. COCO metrics retain detections to confidence 0.001; fixed-threshold metrics use confidence 0.5 and Hungarian mask matching.

#### 3.2.1. Quantitative Comparison

Table 2 reports both COCO-style mask AP and fixed-threshold instance metrics. The four methods are Point-Single, direct Box prompting, Point-Max, and the validation-selected 2×2 Grid-Iter strategy.

AP for small objects is omitted because the COCO evaluator returned −1, indicating that no valid small-object instances were available under the adopted area definition.

The unified results lead to four observations. First, Point-Single is unreliable in dense carton scenes: AP is 0.3031 and F1 is 0.4783. A center point supplies no extent and can activate local texture or an adjacent visible region.

Second, Box is a strong baseline, with AP 0.8984, AP_75_ 0.9438, mIoU 0.9465, and F1 0.9491. The detector boxes already supply effective target extent.

Third, Point-Max raises AP from 0.3031 to 0.8637 and F1 from 0.4783 to 0.9363, showing that candidate selection mitigates point ambiguity. It remains below Box and Grid-Iter because selection cannot add extent when none of the point-generated candidates covers the intended visible surface.

Fourth, Grid-Iter is numerically higher than Box in AP by 0.0005, mIoU by 0.0007, and F1 by 0.0005, but Box is higher in AP_75_ and AR_100_. The paired bootstrap AP difference was 0.0007 (95% CI, −0.0035 to 0.0047), the mIoU difference was 0.0007 (95% CI, −0.0008 to 0.0023; $d_{z}=0.027$), and the F1 difference was 0.0005 (95% CI, −0.0011 to 0.0024; $d_{z}=0.007$). The AP probability that Grid-Iter exceeded Box was 0.611. These intervals include zero, so the evidence does not support overall superiority. The complete paired bootstrap results are summarized in Table 3.

#### 3.2.2. Runtime Efficiency

Table 4 and Table 5 report deployment latency aggregated over three sequential full-test trials. Grid-Iter doubles decoder calls per instance; its mean decoder time (107.54 ms) is therefore about 2.5 times Box (42.88 ms), while the shared 460 ms image encoder masks part of this difference in the total. Point-Max spends 72.85 ms in candidate-selection logic. Peak allocated memory is nearly unchanged because the ViT-H image encoder dominates.

**Figure 6:**
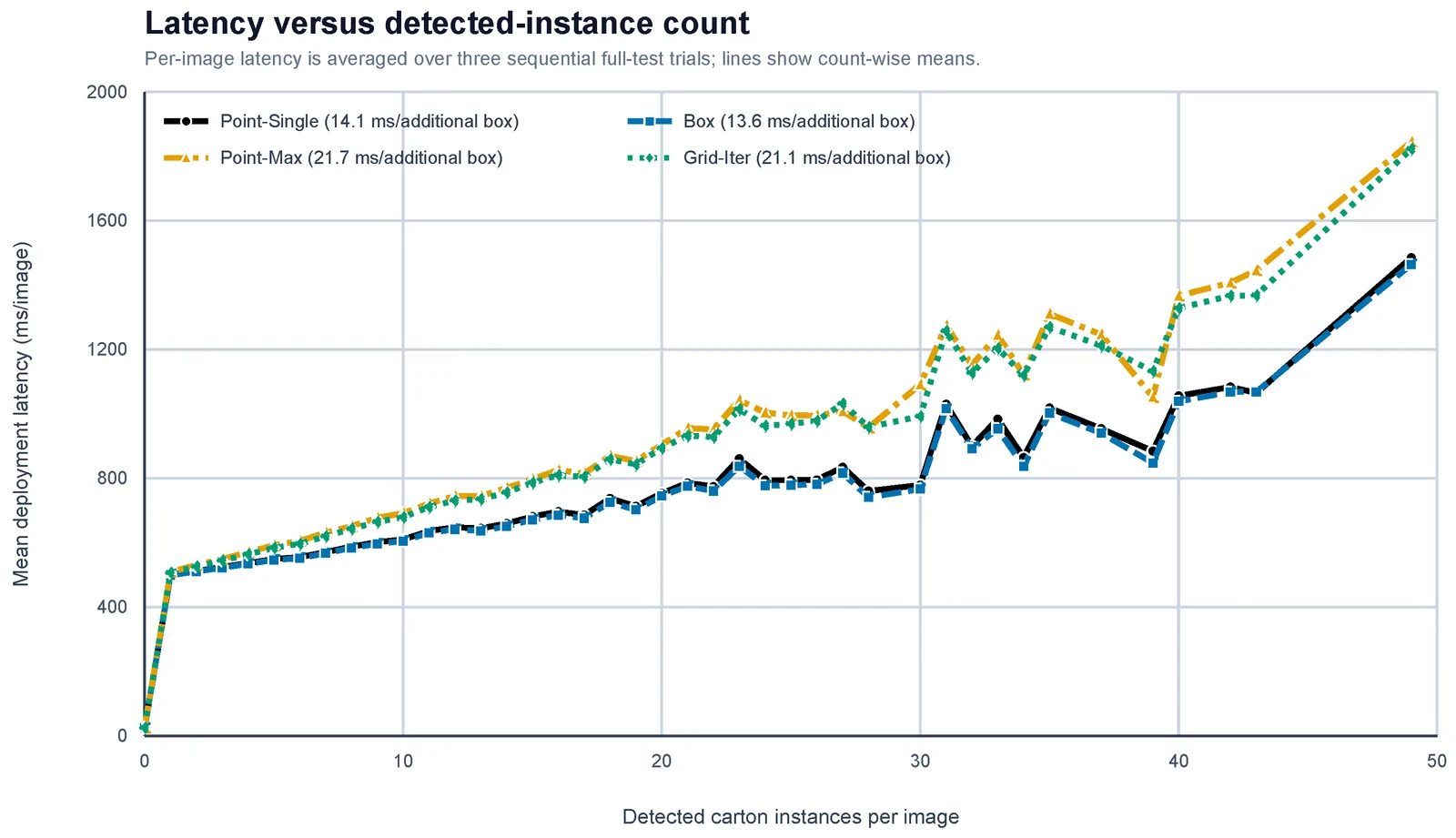
Deployment latency versus the number of detected carton instances. Each point is the mean latency for images with the same count, after first averaging each image over three sequential full-test trials. Parenthetical legend values are ordinary least-squares slopes computed from the 773 per-image means.

Grid-Iter adds 72.66 ms/image relative to Box (paired 95% bootstrap CI, 69.08–76.44 ms; $d_{z}=1.381$), a 12.0% latency increase. Figure 6 further shows that latency rises with detected-instance count. Count-wise linear slopes are 14.1, 13.6, 21.7, and 21.1 ms per additional box for Point-Single, Box, Point-Max, and Grid-Iter, respectively; the corresponding Pearson correlations are 0.911, 0.904, 0.946, and 0.956. Because Grid-Iter’s accuracy intervals include zero, Box is the more economical default. This A10G result does not establish edge-device or real-time readiness.

#### 3.2.3. Boundary-Focused Box–Grid-Iter Comparison

**Figure 7:**
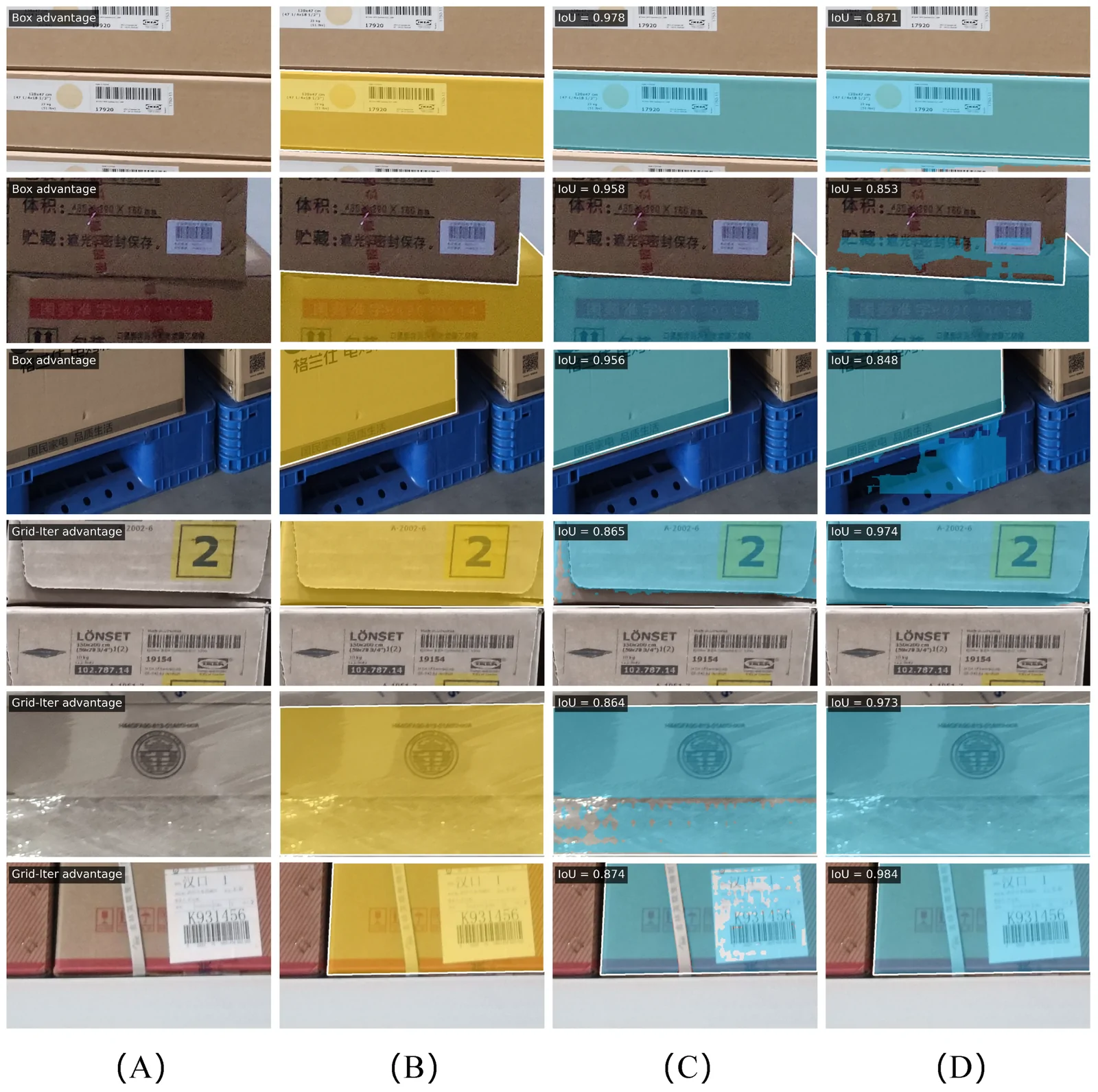
Boundary-focused Box versus Grid-Iter comparison from the formal test set. Columns show (**A**) the RGB crop, (**B**) ground truth, (**C**) Box, and (**D**) Grid-Iter. The first three rows are Box-advantage cases and the final three are Grid-Iter-advantage cases. White contours denote ground truth, yellow denotes its filled region, cyan denotes the prediction, and the displayed values are instance IoUs. Among 7266 comparable instances from all 773 test images, 232 satisfied ΔIoU≤−0.05 and 216 satisfied ΔIoU≥0.05; three representative distinct-image cases per direction were selected by a deterministic script. The examples expose complementary local failures and are not evidence of overall superiority.

Figure 7 provides a boundary-focused comparison that deliberately includes both directions of the Box–Grid-Iter difference. The selection script matched predictions to ground truth over the complete 773-image test set, retained 7266 comparable candidate instances, and formed two pools using |ΔIoU|≥0.05, where $\Delta \mathrm{IoU}={\mathrm{IoU}}_{Grid}-{\mathrm{IoU}}_{Box}$. This produced 232 Box-advantage and 216 Grid-Iter-advantage candidates. Three representative candidates from distinct images were then selected from each pool by the same deterministic rule.

The bidirectional examples are consistent with the paired statistical result. Box can better preserve a target when extra positive grid points fall on a neighboring surface, whereas Grid-Iter can recover a more accurate visible contour when distributed evidence and feedback remain within the intended instance. Neither local behavior dominates the complete test set: the aggregate AP, mIoU, and F1 confidence intervals all include zero.

#### 3.2.4. Four-Strategy Qualitative Overview

**Figure 8:**
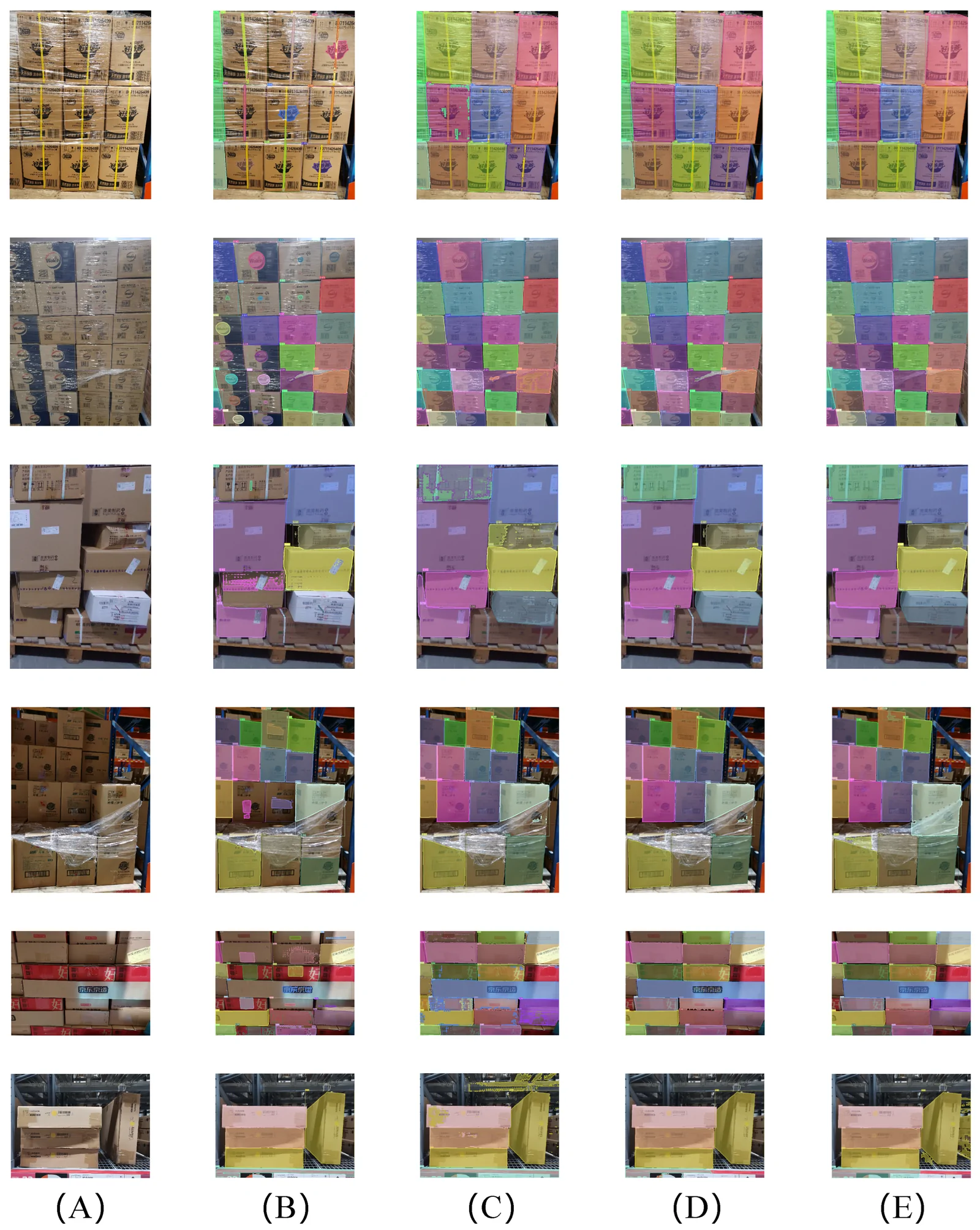
Qualitative comparison of the four prompting strategies across representative visual conditions. The columns show (**A**) RGB image, (**B**) Point-Single, (**C**) Point-Max, (**D**) Box, and (**E**) Grid-Iter. The rows illustrate printed textures, overlapping instances, deformation, reflection, occlusion, and background clutter. White contours denote ground-truth masks.

To place the targeted Box–Grid-Iter boundary analysis in a broader context, Figure 8 compares Point-Single, Point-Max, Box, and Grid-Iter over representative appearance and occlusion conditions. The rows organize qualitative examples rather than acquisition-defined scene groups, and they are used to illustrate characteristic behaviors rather than to estimate performance frequencies.

The overview suggests that a single center point is more vulnerable to local texture and incomplete visible regions, whereas ambiguity-aware candidate selection can mitigate some Point-Single failures. Box and Grid-Iter usually provide stronger spatial guidance, but their complementary boundary failures in Figure 7 and the paired confidence intervals show that neither is uniformly superior.

### 3.3. Validation-Set Prompt Sensitivity and Ablation

The Point-Max selector and grid density were selected on the 773-image validation set, using mask AP as the primary metric and latency or complexity as secondary criteria. The ancillary margin, buffer, kernel, and iteration sweeps were one-factor diagnostic analyses around a 3×3 reference configuration rather than a full factorial search. To avoid post hoc multi-parameter tuning, the prespecified 10% point margin, fixed two-pixel buffer, fixed 5-pixel kernel, and one feedback iteration were retained in the final conceptual Grid-Iter pipeline. Consequently, the complete pipeline is not claimed to be a globally optimized configuration.

#### 3.3.1. Grid Density and Prompt Purity

Table 6 shows that the 2×2 grid has the highest validation AP, mIoU, and F1. Although the fraction of points inside the matched visible mask rises with grid density, the absolute number of incorrectly asserted foreground points also rises from 0.383 per matched instance at 2×2 to 1.514 at 7×7. Thus, the result is not that fewer samples estimate the foreground more accurately. Rather, SAM receives categorical positive prompts, and one additional invalid point can redirect a mask even when the overall point fraction is high.

#### 3.3.2. Ancillary One-Factor Sensitivity

Table 7 reports the requested sensitivity to the point margin, relative buffer, relative morphology kernel, and refinement count. These diagnostic runs used the 3×3 reference configuration, changing one factor at a time; they are therefore not factorially matched to the selected 2×2 test configuration. The adaptive buffer and kernel variants change validation AP by at most 0.0009 relative to their fixed-pixel references. A 20% inward margin is numerically best in its one-factor sweep, and two feedback passes are 0.0002 above one pass, but these settings were not substituted post hoc into the already defined final pipeline. The full Grid-Iter result should thus be read as a conceptual complete interface plus sensitivity analysis, not a claim of global hyperparameter optimality.

#### 3.3.3. Point-Max Candidate Selection

Table 8 compares substantially stronger alternatives than random choice alone. The joint selector has the highest validation AP (0.8750), while box intersection and box coverage have the highest F1 (0.9437). Because AP was predeclared as primary, the joint rule was frozen for the test set. The result supports geometry-aware selection but also shows that candidate ranking remains heuristic and metric dependent.

#### 3.3.4. Grid Component Ablation

The three grid variants were frozen before final evaluation and were run on the identical test split to quantify component effects (Table 9). One feedback decode changes AP by approximately 0.0018 and closing changes AP by less than 0.0001; F1 is slightly higher without either addition. These are small diagnostic differences, not evidence of a causal boundary mechanism.

### 3.4. Cross-Generation Box Reference

Because YOLOv9 and SAM 1 are no longer current, we transferred the simplest interface to a newer pair: a box-only YOLO26s detector and frozen SAM 3. Table 10 reports the result. Both rows use the same cleaned split, test annotations, confidence 0.001 for COCO AP, score 0.5 for fixed metrics, Hungarian matching, and A10G. Model-native differences remain: YOLO26 is end-to-end/NMS-free, SAM 3 uses 1008-pixel input and batched box decoding, and the modern run uses FP16. The table is thus a system-level transfer check, not a claim that the two architectures are capacity- or precision-matched.

YOLO26s–SAM 3 achieved mask AP 0.9115, AP_50_ 0.9783, AP_75_ 0.9578, mIoU 0.9466, precision 0.9466, recall 0.9575, and F1 0.9520. Its five-trial mean was 320.74 ms/image (P95 596.74 ms), versus 604.67 ms/image for the FP32 YOLOv9-C/SAM ViT-H Box system. The transfer supports Box as a stable interface across generations. Speed and accuracy differences cannot be assigned solely to detector or segmenter architecture because precision, preprocessing, batching, and inference heads differ.

### 3.5. Common-Split Supervised Instance-Segmentation References

To contextualize the annotation–accuracy trade-off, Mask R-CNN ResNet-50-FPN-v2, YOLOv8n-seg, and YOLOv9c-seg were trained from polygon masks on the cleaned 6171/773/773 split and exported to the same evaluator. Table 11 compares them with the original detector–SAM Box and Grid-Iter interfaces. Evaluation settings are common: the same 773-image test set, COCO mask AP, at most 300 predictions per image, score 0.5 for fixed-threshold metrics, and Hungarian matching at mask IoU 0.5. Training recipes are model appropriate and were reported in Section 2; the table is therefore a common-data/common-evaluation reference rather than a single-variable architecture ablation.

YOLOv9c-seg obtained the strongest supervised result: mask AP 0.9235, AP_50_ 0.9835, AP_75_ 0.9694, mIoU 0.9504, precision 0.9463, recall 0.9713, and F1 0.9586. YOLOv8n-seg achieved AP 0.9046 and F1 0.9539, while Mask R-CNN achieved AP 0.8517 and F1 0.9129. Thus, the original Box interface is competitive but not uniformly superior to mask-supervised segmentation: it trails YOLOv9c-seg by 0.0251 AP and trails YOLOv8n-seg by 0.0062 AP. The appropriate interpretation is an annotation trade-off. The detector–SAM systems avoid carton-specific pixel-mask training of the segmenter, whereas the supervised models consume the available polygons during fitting.

### 3.6. Scene-Stratified Analysis

To probe within-dataset robustness without claiming external generalization, images were divided by carton count, brightness tertile, and a background edge-density proxy using thresholds fixed without outcome inspection. Table 12 reports representative density strata. Box and Grid-Iter remain close in every stratum. Both obtain lower F1 in low-density images because a small number of false or missed instances has a larger proportional effect; high-density images increase computational load but do not reverse the main accuracy conclusion. Edge density and visible-area ratio are proxies rather than direct causal measurements of clutter or occlusion.

## 4. Discussion

The central result is a trade-off, not a universally superior prompt. Point-Max demonstrates that ambiguity-aware selection recovers much of the performance lost by an under-specified center point, but it cannot add spatial evidence absent from all point-generated candidates. Box communicates extent directly and has the fewest interface operations. Grid-Iter distributes positive evidence and adds a second decoder call, but it also creates invalid-positive risk and additional hyperparameters.

The paired intervals explain why methods should not be ranked solely by their fourth decimal place. Grid-Iter is numerically higher than Box in AP, mIoU, and F1, but every paired accuracy interval includes zero; Box is higher in AP_75_ and AR_100_ and is 12.0% faster. We therefore recommend Box as the default. Grid-Iter remains a conditional option only when an application validates a meaningful contour gain and can absorb the extra decoder cost.

The grid-purity analysis refines the explanation for the smaller grid. Denser grids contain a larger fraction of valid points but also more invalid foreground assertions in absolute terms. This association is consistent with prompt contamination but does not prove that invalid points alone cause the AP decline. Likewise, the small logit-feedback and morphology ablations do not support strong causal claims about attention, boundary recovery, topology repair, or overfitting.

The YOLO26s–SAM 3 reference shows that Box transfers to a newer system and improves the system-level AP/latency point. It neither makes the original components current nor isolates which modern component caused the change. One of five timing trials contained substantially more host-side outliers than the other four, which increased the aggregate standard deviation and P95; all trials are retained rather than selectively discarded. A controlled generational study would need to match capacity, precision, input transforms, batching, detection heads, and training recipes.

The common-split supervised references clarify the scope of the annotation claim. YOLOv9c-seg is numerically strongest (AP 0.9235 and F1 0.9586), followed by the modern YOLO26s–SAM 3 Box system in AP (0.9115). The original YOLOv9-C/SAM Box interface is slightly below YOLOv8n-seg in AP but close in matched-mask quality. Consequently, the detector–promptable-segmenter design is not presented as an accuracy replacement for all supervised segmenters. Its advantage is that carton-specific adaptation of the segmentation stage uses boxes rather than polygon masks; the detector still needs box labels, and this study still needs polygons for prompt selection and evaluation.

Limitations include detector error propagation, one public dataset, the absence of authoritative acquisition-scene identifiers, two filename-derived cross-split group candidates, heuristic Point-Max/Grid-Iter components, model-specific supervised training recipes, and hardware-dependent latency. The near-duplicate audit removes a verified leakage source but cannot prove complete scene independence. Scene-stratified outputs by instance density, visible area, brightness, and edge clutter narrow claims to the observed SCD range; they cannot replace testing at an independent warehouse. The masks represent visible surfaces, not amodal carton geometry. Future work should evaluate grouped acquisition splits, external sites, additional lightweight promptable segmenters, learned prompt routing, and depth or multi-view geometry. Real-time and edge claims require tests on the actual target device and a declared throughput requirement.

## 5. Conclusions

This study recasts YOLO–SAM carton segmentation as a training-free detector-to-promptable-segmenter interface problem. On 773 leakage-audited test images, Box and validation-selected 2×2 Grid-Iter achieved mask AP 0.8984 and 0.8988, mIoU 0.9465 and 0.9472, and F1 0.9491 and 0.9496. Their paired accuracy intervals included zero, while Grid-Iter added 72.66 ms/image (95% CI, 69.08–76.44 ms). Box is therefore the parsimonious default, and Grid-Iter is a conditional alternative.

Point-Max confirms that a stronger candidate selector mitigates single-point ambiguity, and the prompt-purity study explains why more positive points need not improve a promptable segmenter. The YOLO26s–SAM 3 Box reference reached mask AP 0.9115 and 320.74 ms/image, supporting transfer of the interface conclusion to newer components under disclosed model-native settings. Common-split supervised references further showed AP 0.8517 for Mask R-CNN, 0.9046 for YOLOv8n-seg, and 0.9235 for YOLOv9c-seg. These results position the proposed interface as an annotation–accuracy option rather than a universal accuracy winner. The contribution is an uncertainty-aware accuracy–latency decision framework and reproducible evaluation protocol, not the mere combination of YOLOv9 and SAM.

## Figures and Tables

**Table 1:** Detection performance of YOLOv9 on the SCD test set.

Detector	Box AP	AP_50_	AP_75_	AR_100_
YOLOv9-C	0.9026	0.9703	0.9445	0.9278

**Table 2:** Unified YOLOv9-C/SAM ViT-H comparison on the 773-image SCD test set. COCO metrics retain low-score predictions; fixed-threshold metrics use Hungarian matching at mask IoU ≥0.5. Bold values denote numerical, not necessarily statistically significant, best values.

Strategy	Mask AP	AP_50_	AP_75_	AR_100_	mIoU	Precision	Recall	F1
Point-Single	0.3031	0.3618	0.3132	0.5090	0.8993	0.4890	0.4681	0.4783
Box	0.8984	0.9690	**0.9438**	**0.9241**	0.9465	0.9702	0.9289	0.9491
Point-Max	0.8637	0.9495	0.9108	0.9016	0.9411	0.9571	0.9163	0.9363
Grid-Iter (2×2)	**0.8988**	**0.9702**	0.9388	0.9238	**0.9472**	**0.9707**	**0.9294**	**0.9496**

**Table 3:** Paired bootstrap comparison, Grid-Iter minus Box.

Metric	Difference	95% CI	$d_{z}$	p(Δ>0)
Mask AP	0.0007	[−0.0035, 0.0047]	—	0.611
Matched mIoU	0.0007	[−0.0008, 0.0023]	0.027	0.827
F1	0.0005	[−0.0011, 0.0024]	0.007	0.674

**Table 4:** Synchronized latency distribution on one NVIDIA A10G, aggregated over three trials (2319 timed observations per strategy). Times are milliseconds per image and exclude image loading, serialization, visualization, and evaluation. Bold values denote the minimum values in the corresponding latency-distribution columns.

Strategy	Mean	Std.	P50	P95	Peak MB
Point-Single	610.16	107.67	583.10	808.62	6131.5
Box	**604.67**	**104.26**	**577.79**	**791.21**	6131.5
Point-Max	687.93	159.15	649.63	997.02	6131.5
Grid-Iter (2×2)	677.33	153.19	642.11	972.55	6131.5

**Table 5:** Mean synchronized component time in milliseconds per image. Post-processing includes scaling masks and boxes back to annotation coordinates.

Strategy	YOLO	SAM Encoder	SAM Decoder	Logic	Post-Processing
Point-Single	26.99	460.06	47.81	0.99	74.31
Box	26.89	460.10	42.88	1.00	73.80
Point-Max	26.92	459.89	53.33	72.85	74.93
Grid-Iter (2×2)	26.93	460.09	107.54	8.19	74.59

**Table 6:** Validation-set sensitivity of Grid-Iter to grid density. Bold values denote the numerically best values for the corresponding metrics; higher values are preferred except for Invalid Points/Instance, for which lower values are preferred.

Grid	Mask AP	mIoU	F1	Prompt Purity	Invalid Points/Instance
2×2	**0.9050**	**0.9468**	**0.9548**	0.9042	**0.383**
3×3	0.8986	0.9448	0.9530	0.9415	0.526
5×5	0.8935	0.9426	0.9517	0.9623	0.943
7×7	0.8875	0.9401	0.9515	**0.9691**	1.514

**Table 7:** One-factor Grid-Iter sensitivity on the validation set using a 3×3 reference grid. Values inside parentheses are mask AP in the same order as the listed settings.

Factor	Settings and Mask AP	Prespecified Final Value	Interpretation
Point margin	0.05/0.10/0.15/0.20 (0.8951/0.8986/0.9017/0.9022)	0.10	diagnostic trend
Buffer ratio	0/0.005/0.01/0.02 (0.8986/0.8990/0.8987/0.8989)	fixed 2 pixels	negligible spread
Kernel ratio	0/0.01/0.02/0.04 (0.8986/0.8986/0.8986/0.8995)	fixed 5 pixels	negligible spread
Feedback passes	0/1/2 (0.8925/0.8986/0.8988)	1	one-pass Pareto choice

**Table 8:** Validation-set Point-Max selector comparison. Bold values denote the numerical best values in each metric column; tied best values are both shown in bold.

Selector	Mask AP	mIoU	F1
Seeded random	0.3803	0.9057	0.6234
SAM score	0.4748	0.9359	0.6783
Box intersection	0.8561	0.9338	**0.9437**
Box coverage	0.8561	0.9338	**0.9437**
Geometric maximum	0.8729	0.9406	0.9431
Joint score	**0.8750**	**0.9424**	0.9406

**Table 9:** Grid-family component ablation on the common test split. Bold values denote the numerical best values in each column; tied best values are both shown in bold.

Configuration	Mask AP	mIoU	Precision	Recall	F1
Grid, no feedback	0.8971	0.9461	**0.9717**	**0.9303**	**0.9505**
Grid-Iter, no morphology	**0.8988**	0.9467	0.9709	0.9295	0.9497
Grid-Iter, complete	**0.8988**	**0.9472**	0.9707	0.9294	0.9496

**Table 10:** Cross-generation transfer of direct Box prompting. Latency is hardware-specific; system settings are disclosed rather than treated as a single-variable ablation. Bold values denote the numerical best values, with higher values preferred for accuracy metrics and lower values preferred for latency.

System	Compute Mode	Mask AP	AP_50_	mIoU	F1	Mean ms
YOLOv9-C + SAM ViT-H	FP32	0.8984	0.9690	0.9465	0.9491	604.67
YOLO26s + SAM 3	FP16	**0.9115**	**0.9783**	**0.9466**	**0.9520**	**320.74**

**Table 11:** Common-split supervised instance-segmentation context. “Pixel-mask training” indicates whether carton polygon masks updated the segmentation model. Polygon masks were used for validation and evaluation for every row. Bold values denote numerical best values.

System	Pixel-Mask Training	Mask AP	AP_50_	AP_75_	AR_100_	mIoU	F1
YOLOv9-C + SAM Box	No	0.8984	0.9690	0.9438	0.9241	0.9465	0.9491
YOLOv9-C + SAM Grid-Iter	No	0.8988	0.9702	0.9388	0.9238	0.9472	0.9496
Mask R-CNN R50-FPN-v2	Yes	0.8517	0.9178	0.8964	0.8798	0.9473	0.9129
YOLOv8n-seg	Yes	0.9046	0.9790	0.9544	0.9268	0.9469	0.9539
YOLOv9c-seg	Yes	**0.9235**	**0.9835**	**0.9694**	**0.9410**	**0.9504**	**0.9586**

**Table 12:** Test performance stratified by carton density.

Strategy	Density Stratum	Images	mIoU	F1
Box	Low (≤5)	197	0.9500	0.9151
Box	Medium (6–10)	318	0.9500	0.9469
Box	High (>10)	258	0.9440	0.9557
Grid-Iter	Low (≤5)	197	0.9539	0.9109
Grid-Iter	Medium (6–10)	318	0.9513	0.9501
Grid-Iter	High (>10)	258	0.9439	0.9555

## Data Availability

The Stacked Carton Dataset is publicly available as described in [1]. The cleaned split contains 6171 training, 773 validation, and 773 test images. The complete version 1.0.0 reproducibility suite is permanently archived on Zenodo at https://doi.org/10.5281/zenodo.21860345 [37]. It contains the frozen code and configurations, cleaned split manifests, derived COCO annotations, author-trained detector and supervised-baseline checkpoints, prediction files, per-image timing measurements, statistical analyses, and figure-audit materials. Dataset images are not redistributed and remain subject to the original SCD license. Third-party foundation-model checkpoints are also excluded; the archive documents their official sources, versions, access conditions, and available integrity identifiers. File-level integrity is recorded in SHA256SUMS.txt.

## Abbreviations

The following abbreviations are used in this manuscript:

AP	Average Precision
AR	Average Recall
CIoU	Complete Intersection over Union
DFL	Distribution Focal Loss
F1	F1-score
GELAN	Generalized Efficient Layer Aggregation Network
IoU	Intersection over Union
mIoU	Mean Intersection over Union
NMS	Non-Maximum Suppression
PGI	Programmable Gradient Information
ROI	Region of Interest
SAM	Segment Anything Model
SCD	Stacked Carton Dataset
YOLO	You Only Look Once
